# Molecular Mechanisms Involving the Sonic Hedgehog Pathway in Lung Cancer Therapy: Recent Advances

**DOI:** 10.3389/fonc.2022.729088

**Published:** 2022-04-01

**Authors:** Chao Ma, Kang Hu, Irfan Ullah, Qing-Kang Zheng, Nan Zhang, Zhi-Gang Sun

**Affiliations:** ^1^ School of Clinical Medicine, Weifang Medical University, Weifang, China; ^2^ Department of Thoracic Surgery, Central Hospital Affiliated to Shandong First Medical University, Jinan, China; ^3^ Department of Surgery, Khyber Medical University Peshawar, Peshawar, Pakistan; ^4^ Breast Center, Central Hospital Affiliated to Shandong First Medical University, Jinan, China

**Keywords:** lung cancer, Hh pathway, proliferation, invasion, metastasis, natural product

## Abstract

According to the latest statistics from the International Agency for Research on Cancer (IARC), lung cancer is one of the most lethal malignancies in the world, accounting for approximately 18% of all cancer-associated deaths. Yet, even with aggressive interventions for advanced lung cancer, the five-year survival rate remains low, at around 15%. The hedgehog signaling pathway is highly conserved during embryonic development and is involved in tissue homeostasis as well as organ development. However, studies have documented an increasing prevalence of aberrant activation of HH signaling in lung cancer patients, promoting malignant lung cancer progression with poor prognostic outcomes. Inhibitors targeting the HH pathway have been widely used in tumor therapy, however, they still cannot avoid the occurrence of drug resistance. Interestingly, natural products, either alone or in combination with chemotherapy, have greatly improved overall survival outcomes for lung cancer patients by acting on the HH signaling pathway because of its unique and excellent pharmacological properties. In this review, we elucidate on the underlying molecular mechanisms through which the HH pathway promotes malignant biological behaviors in lung cancer, as well as the potential of inhibitors or natural compounds in targeting HH signaling for clinical applications in lung cancer therapy.

## Introduction

Lung cancer, which is one of the most lethal malignancies worldwide, can be classified into non-small cell lung cancer (NCSLC) or small cell lung cancer (SCLC) based on histopathological type (1–3). Among them, NSCLC accounts for approximately 85% of lung cancer cases (4–6). Regrettably, most lung cancer patients are already in advanced stages at the time of diagnosis, losing the opportunity for surgery. However, with decades of medical development, diverse and individualized treatment strategies for advanced lung cancer, including chemotherapy, radiotherapy, targeted therapy as well as immunotherapy have been developed (7, 8). Among them, molecular targeted therapies targeting driver genes has shown good application prospects, and the corresponding molecular targeted drugs have been developed for common lung cancer driver genes, such as epidermal growth factor receptor (EGFR), mesenchymal lymphoma kinase (ALK) and c-ros oncogene 1 receptor tyrosine kinase (ROS1), significantly prolonging progression-free survival (PFS) and overall survival (OS) outcomes for lung cancer patients (9–11).

The HH signaling pathway is highly evolutionarily conserved, and maintains tissue homeostasis and organ development (12, 13). There is evidence that HH signaling participates in embryonic lung development, regulates epithelial-mesenchymal interactions, differentiation of embryonic neuroendocrine cells, and repair of injured airway tissues (14). Aberrant activation of HH signaling is closely associated with lung cancer development (15). The Kras/YY1/ZNF322/SHH transcriptional axis increases the expression levels of the SHH protein, leading to lung cancer malignant progression by inducing tumor angiogenesis (16).

In lung squamous cell carcinoma (LSCC), HH signaling regulates EMT-associated proteins, such as E-Cadherin and β-catenin to induce the EMT process (17), and Smo gene amplification is one of the mechanisms through which HH signaling is activated to increase the resistance of lung cancer cells to epidermal growth factor receptor tyrosine kinase inhibitors (EGFR-TKIs) by inducing the EMT process (18). In an EGFR-TKIs-resistant lung cancer mouse model, the HH inhibitor, LDE225 (Sonidegib), in combination with gefitinib significantly inhibited tumor growth than gefitinib alone, completely inhibiting the phosphorylation of PI3K/AKT and MAPK signaling. Moreover, LDE225 enhanced the sensitivity of lung cancer cells to standard chemotherapy (18). In addition, to establish whether HH signaling was activated in lung cancer cells, Gli1 protein levels in advanced NSCLC were assessed. Gli1 protein overexpression was found to be associated with poor prognostic outcomes and immune checkpoint inhibitor resistance (19). Activation of HH signaling is essential for the development of some types of SCLC (14). In an SCLC mouse model, inhibition of HH signaling using cyclopamine significantly reduced tumor growth. HH inhibitors in combination with radiotherapy or chemotherapy regimens provide individualized treatment options for SCLC patients (14).

In this review, we elucidate on the underlying molecular mechanisms of HH signaling in lung cancer development and explore the relevance of HH signaling-related proteins in the prognosis of several types of lung cancer, indicating new directions for research.

## The Canonical Pathway of HH Signaling

The hedgehog (HH) gene was first detected in 1980 by Nüsslein-Volhard and Wieschaus while studying the embryonic development of Drosophila (20). As the canonical signaling pathway in vertebrates, the HH-Ptch-Smo-Gli route consists of several essential components, including Sonic hedgehog (SHH), Desert hedgehog (DHH), and Indian hedgehog (IHH), Smoothened (Smo), Patched (Ptch), three glioma-associated oncogene transcription factors (Gli1, Gli2, Gli3), Suppressor of fused (SuFu). Of the three HH homologs, the SHH protein is highly expressed, with the most potent biological effects (21). Smo, a seven-pass transmembrane protein belonging to the G protein-coupled receptor (GPCR) superfamily, can be inhibited by Ptch. Ptch, a twelve-pass transmembrane protein to be the receptor of SHH, can negatively regulate the HH pathway. As downstream effectors of the HH signaling pathway, the Gli zinc-finger transcription factor family plays a crucial role in final activation or inactivation of the HH pathway (22). Gli1 acts as a transcriptional activator because it lacks an N-terminal inhibitory region, while Gli2 and Gli3 both have N-terminal inhibitory regions and C-terminal activation regions that play dual roles of activation and repression (23). The Gli1 protein acts as a key transcription factor, regulating the expressions of downstream target genes of the HH pathway.

In the absence of HH ligands, Ptch receptors constitutively inhibit the activities of Smo proteins (24), leading to the phosphorylation of Gli proteins (Gli2 and Gli3) within the microtubule complex by casein kinase 1a (CK1a), protein kinase A (PKA), and glycogen synthase kinase 3β (GSK3β). The phosphorylated Gli proteins then bind β-transducing repeat-containing protein (β-TrCP) to form the Gli/β-TrCP complex that is sheared into a transcription-repressive form (Gli-R) by the actions of ubiquitin-proteasome (25). Briefly, when the HH signaling pathway is inactivated, Gli-R enters the nucleus, where they bind target gene promoters to repress gene transcriptions.

**Figure 1:**
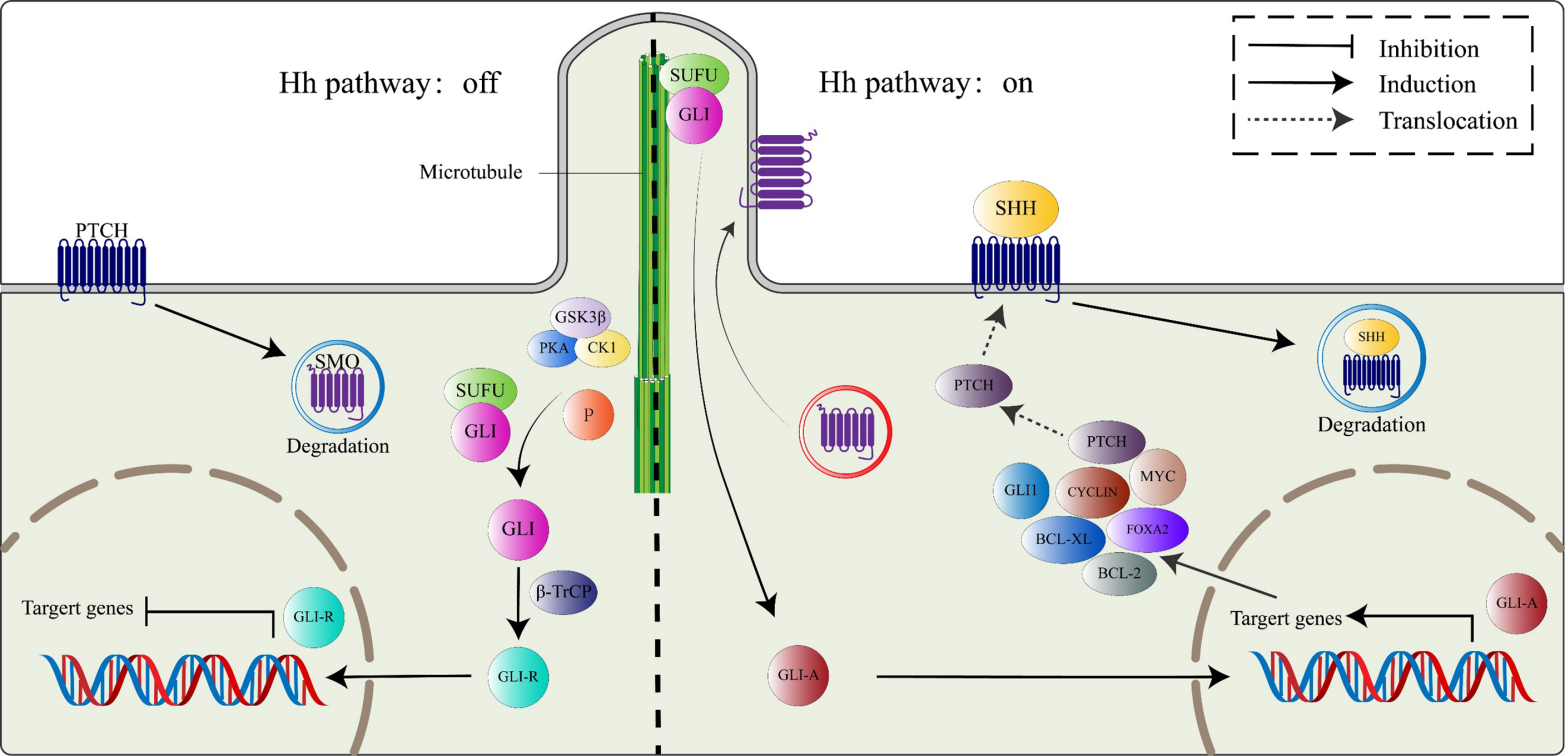
The canonical HH signaling pathway. In the absence of the HH ligand (left), PTCH binds SMO, leading to its degradation. Microtubule complex containing Gli and SUFU is phosphorylated by GSK3β/PKA/CK1 to activate Gli proteins, which subsequently combine with β-TrCP to switch into transcriptional repressor (GLI-R). In the presence of HH ligand (right), SMO restriction by PTCH is relieved, active SMO moves to the primary cilium. Active Smo interacts with the SUFU/GLI complex, localized at the top of the primary cilium, GLI proteins from the complex switch into transcription activators (GLI-A), which then translocate to the nucleus and activate the HH target genes, including PTCH, GLI1, FOXA2, BCL-2, BCL-Xl, MYC, and CYCLIN family among others.

In the presence of HH ligands, inhibitory effects of Ptch receptors on Smo proteins are alleviated. The Smo protein, activated by CK1, PKA, and GPCR kinase 2 (GRK2)-mediated phosphorylation, translocates to the primary cilium by interacting with β-arrestin (26). As Smo proteins continuously accumulate on the primary cilium, a microtubule complex consisting of Gli and SuFu proteins translocate to the top of the primary cilium (27), where it inhibits Gli proteins C-terminal hydrolysis by interacting with Smo proteins, thereby activating and releasing Gli proteins. The activated Gli protein (Gli-A), which has a transcriptional activation function, enters the nucleus to regulate the expressions of HH pathway target genes (28). Target genes of the HH pathway include Gli1, Ptch1, FoxA2, Bcl-2, Bcl-xl, Myc, and Cyclin family (**Figure 1**) (29).

## The Noncanonical Pathway of HH Signaling

**Figure 2:**
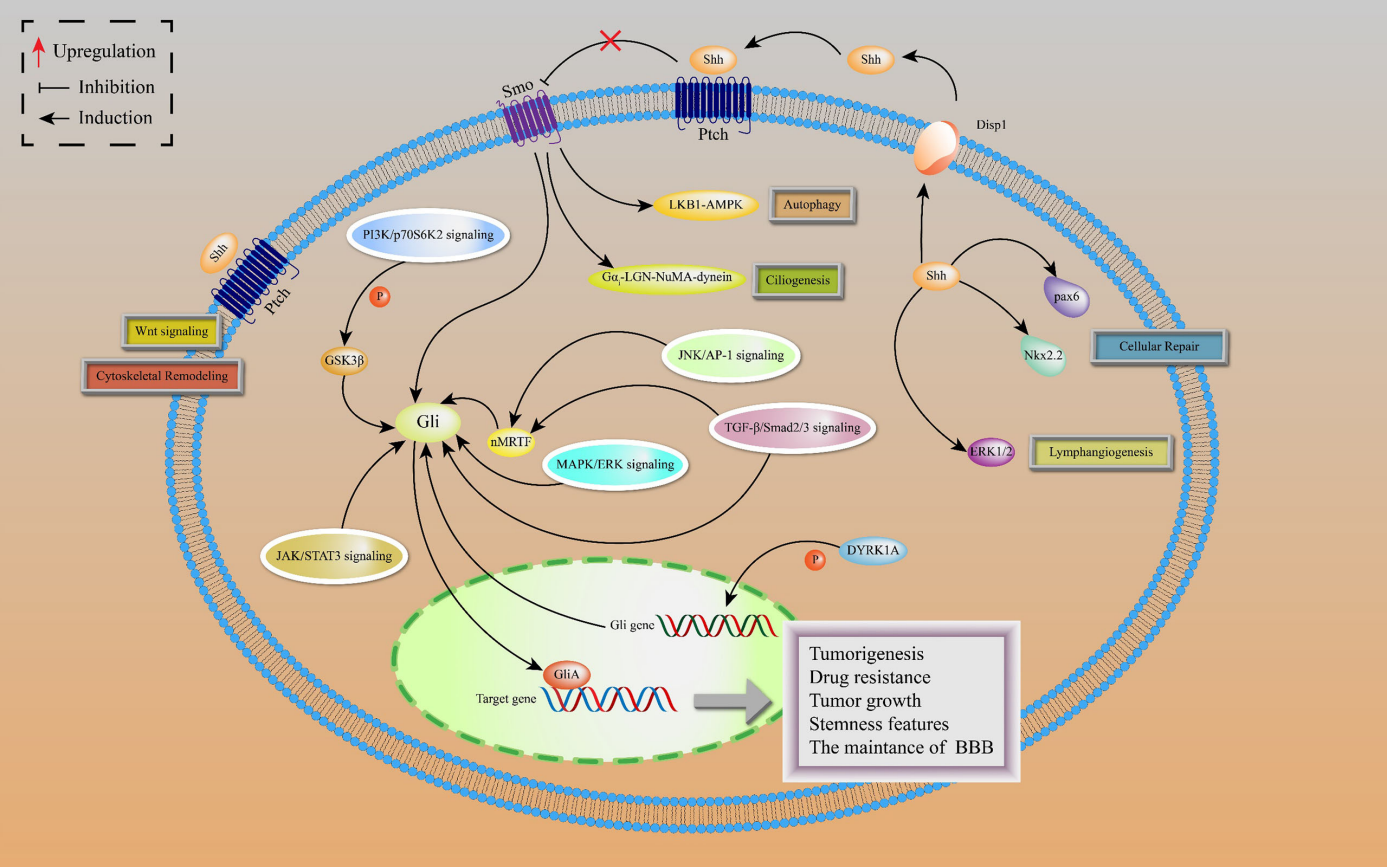
Noncanonical HH signaling transductions are depicted, including SHH-mediated, Ptch-mediated, Smo-mediated and Gli-mediated noncanonical signaling. SHH-mediated noncanonical pathway is associated with cellular repair and lymphangiogenesis. Ptch-mediated but Smo-independent pathway is activated by SHH to induce Wnt signaling and cytoskeletal remodeling. In Smo-mediated but Gli-independent noncanonical pathway, the SHH ligand activates LKB1-AMPK and Gαi-LGN-NuMA-dynein axes in neurons, leading to activation of autophagy and ciliogenesis. There is a crosstalk between Gli activated and Smo-independent pathways as well as with numerous oncogenic pathways, resulting in lung cancer tumorigenesis and development.

Noncanonical HH signal transduction is the signaling response to any associated components of the HH pathway, different from the common canonical HH signaling pathway. Based on HH pathway-related components, the noncanonical HH pathway is classified into four types: SHH-mediated, Ptch-mediated, Smo-mediated, and Gli-mediated noncanonical signaling (**Figure 2**).

The SHH ligands selectively promote lymphatic endothelial cell proliferation to induce lymphangiogenesis after kidney injury by activating extracellular signal-regulated kinase-1 and -2 (ERK1/2) pathway, leading to kidney fibrosis, independently of Smo (30). Moreover, SHH proteins are involved in repairing responses of damaged cerebellum by upregulating the expressions of Nkx2.2 and Pax6 transcription factors in cadmium-exposed rats (31).

The Ptch-mediated noncanonical pathway is a Ptch-mediated Smo-independent pathway. This pathway is accompanied by cytoskeletal remodeling and Wnt signal activation, which provides a possible explanation for the failure of Smo inhibitors in HH-dependent malignant tumors (32). In addition, active Smo induces autophagy and promotes ciliogenesis by acting on LKB1-AMPK and Gαi-LGN-NuMA-dynein axes in some cell lines, including neurons, independently of the Smo-mediated canonical pathway (33).

The Gli-mediated noncanonical pathway is also known as Gli-activated, but Smo-independent pathway. In drug-resistant basal cell carcinoma (BCC), activator protein-1 (AP-1) and transforming growth factor-β (TGFβ) synergistically stimulate a nuclear myocardin-related transcription factor (nMRTF) to enhance transcriptional activities of Gli1 (34). Desmoglein 2 activates signal transducer and activator of transcription 3 (STAT3) to upregulate Gli1 expressions, resulting in BCC occurrence and development (35). Astrocyte-derived TGFβ1/Smad2/3 signaling contributes to blood-brain barrier (BBB) functions by increasing ZO-1 expression *via* upregulating Gli2 protein levels (36). In addition, dual-specificity tyrosine-regulated kinase 1A (DYRK1A) phosphorylate Gli1 at Ser408 to enhance the transcriptional activities of Gli1, suggesting that DYRK1A regulates noncanonical HH signaling (37).

**Figure 3:**
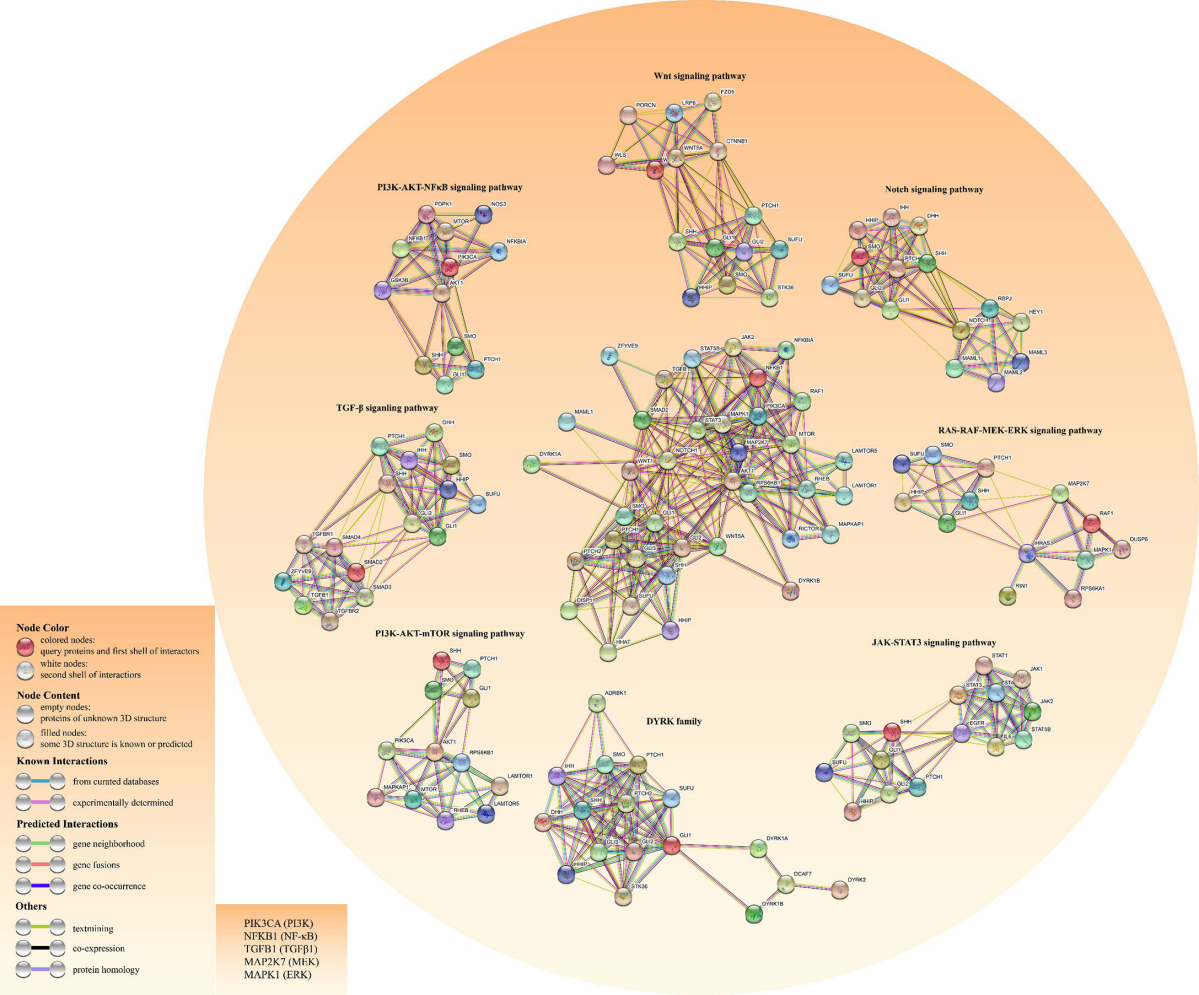
Construction of the crosstalk network for both HH pathway and other cancer-related pathways using the STRING database. The left bottom literal statements are the alternative names of the molecule.

In lung adenocarcinoma (LAC), KRAS and vascular endothelial growth factor (VEGF) receptor, NRP2, trigger MAPK/ERK/Gli1 signaling cascade to promote tumor progression (38). As a downstream effector of the PI3K pathway, p70S6K2 enhances the transcription activities of Gli1 by modulating the phosphorylation of GSK3β in NCSLC (39). Finally, there are other oncogenic pathways involving noncanonical HH signal transduction, such as AMP-activated protein kinase (AMPK), PI3K-AKT-mTOR signaling, and Protein kinase C (PKC) (40). **Figure 3** shows the crosstalk between the HH pathway with various oncogenic pathways.

## The HH Pathway Promotes the Malignant Progression of Lung Cancer

Abnormal activations of the HH signal are closely associated with lung cancer occurrence and development. They are involved in cell proliferation, invasion, metastasis, drug resistance, stemness characteristics, and tumor microenvironment. Therefore, we summarize the recent findings of HH signaling in lung cancer and discuss the potential for targeting the HH pathways to treat lung cancer.

### The HH Pathway Promotes Lung Cancer Cell Proliferation

The HH pathway remarkably induces tumor proliferation (41, 42). HH signaling regulates INSM1 interactions with N-myc to promote SCLC cell growth in a Gli-dependent manner (43). The E3 ligase (HERC4) and liver kinase B1 (LKB1) inhibit cell proliferation in lung cancer by inactivating the HH pathway. Mechanistically, HERC4 has been shown to negatively regulate the HH pathway by destabilizing the Smo protein (44), while the mechanisms through which the HH pathway is inhibited by LKB1 have not been elucidated (45).

### HH Pathway Promotes Lung Cancer Cell Invasion and Migration

The HH pathway is involved in cell invasion and migration of multiple tumors (46). SAM- and SH3-domain containing 1 (SASH1) inhibit hepatocellular carcinoma invasion and migration by inactivating the HH and PI3K/AKT pathways *in vitro and in vivo* (47). *In vitro*, transmembrane 107 protein (TMEM107) inhibited epithelial-mesenchymal transition (EMT) and invasion by negatively regulating HH signaling in NSCLC (48). Besides, Gli1, the downstream gene of the HH pathway, promotes NSCLC cell invasion and metastasis *in vitro* and *in vivo* by inducing the EMT process. The downregulation of Gli1 significantly inhibited tumor growth and enhanced E-cadherin expressions (49). Interestingly, the KRAS mutation activated the Kras/YY1/ZNF322A/SHH axis by triggering the expression of downstream genes, including YY1, ZNF322A, and SHH, which promoted angiogenesis in NSCLC *in vitro and in vivo* (16). The overexpression of phosphatidylethanolamine‐binding protein 4 (PEBP4) promotes NSCLC cell proliferation and EMT by regulating the HH pathway (50) while fibroblast activation protein α (FAPα) promoted LSCC cell growth, adhesion, and migration *in vitro via* the PI3K and HH pathways (51).

Hedgehog-interacting proteins (HHIP) negatively regulate the HH pathway by binding SHH proteins (52–54). HHIP serve as tumor suppressors by significantly inhibiting lung cancer cell proliferation, migration, and invasion (53). Similarly, overexpression of signal peptide-CUB-EGF domain-containing protein 2 (SCUBE2) inhibits NSCLC cell proliferation and invasion by modulating the HH pathway. The SCUBE2-mediated inhibition of the HH pathway can be reversed by recombinant SHH proteins (55). GATA-6 impairs the metastatic abilities of LAC cells (56). Moreover, GATA-6 suppresses cell proliferation and migration in LSCC by inhibiting the expressions of SHH at transcriptional levels (57). Additionally, miR‐520b enhances NSCLC cell proliferation and metastasis by activating the SPOP‐Gli2/3 axis (58). Hematopoietic pre-B-cell leukemia transcription factor (PBX)-interacting protein (HPIP) is a nucleo-cytoplasmic shuttling protein (59) that promotes NSCLC cell proliferation, invasion, and migration *in vitro* and tumor growt*h in vivo* by interfering with the HH pathway (54).

**Figure 4:**
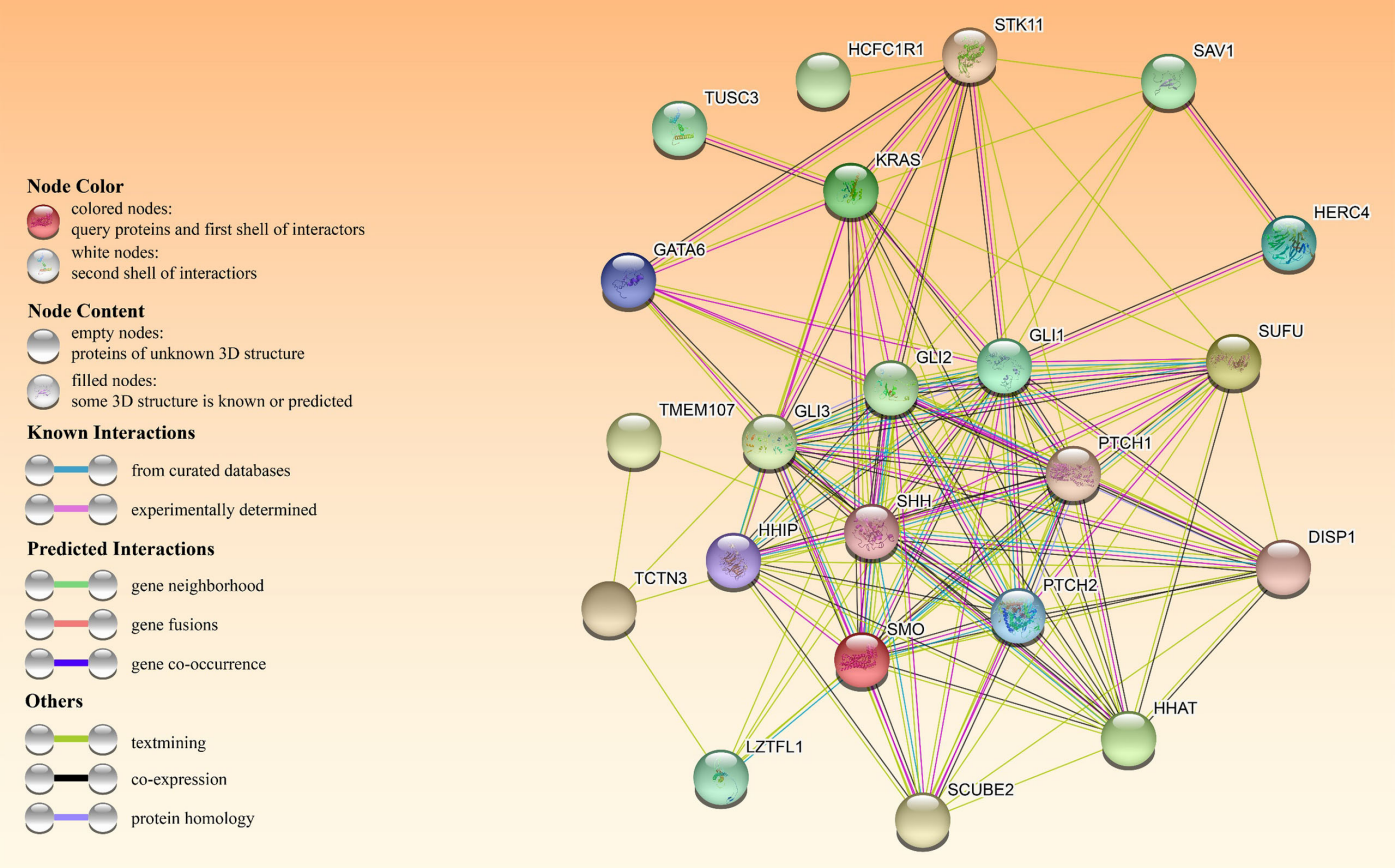
The HH pathway interacts with other oncogenes or tumor suppressors in patients with HH-dependent lung cancer. The interactome in the center was obtained through the STRING database.

In an A549 cell line, tumor suppressor candidate 3 silencing (TUSC3) inhibited growth, proliferation and induced apoptosis as well as radiation sensitivity (60). In contrast, TUSC3 overexpression in NSCLC promoted cell proliferation, migration, and invasion *in vitro* and *in vivo*, possibly by involving HH signaling (61). Leucine zipper transcription factor-like 1 (LZTFL1) inhibits lung cancer tumorigenesis, at least partly by inhibiting HH signaling pathway to maintain epithelial cell differentiation (62). **Figure 4** shows HH signaling interaction networks in lung cancer patients.

### HH Pathway and Drug Resistance

Drug resistance is one of the leading causes of tumor treatment failure (63), while hyperactivation of the HH pathway is frequently described in many drugs resistant malignant tumors. The HH pathway promotes cell proliferation as well as invasion in LAC with acquired drug resistance to EGFR-TKIs, partially by acting on HGF and MET signaling (64). Della Corte et al. (65) investigated the mechanisms in acquired resistance to EFGR-TKI during NSCLC treatment. The HH pathway was found to induce drug resistance by regulating the EMT process. Additionally, the active HH pathway was shown to induce EMT and upregulated ABCG2 in EGFR-TKI-resistant NSCLC patients, while the opposite result was observed when the HH pathway was inhibited (66).

MiRNA disorders are prevalent in many malignant tumors, including lung cancer (67, 68). miR-182-5p is the direct target of Gli2, the negative feedback axis of miR-182-5p/Gli2 regulates cell cisplatin resistance in LAC (69).

### HH Pathway and Lung Cancer CSCs

A small proportion of tumor cells are regarded as CSCs that play a vital role in tumor occurrence, development and recurrence (70, 71). Aberrantly HH signaling is common in CSCs (72, 73). SHH-positive (SHH^+^) cells in NSCLC, expressing an uncleaved full-length SHH protein, exhibited drug resistance and CSC characteristics. A high abundance of SHH^+^ cells is a biomarker for worse prognostic outcomes for NSCLC patients (74). Low-folate (LF) is associated with malignant progression of lung cancer. Functionally, the LF microenvironment increases CSCs-like potential, partially through the HH pathway (75). Fibroblast growth factor receptor 1 (FGFR1) amplification in NSCLC is particularly prevalent, especially in LSCC (76, 77), where it stimulates the expressions of Gli2 through ERK pathway activation. The FGFR1/Gli2 axis upregulates the expression levels of CSCs markers (78). Aberrantly activated Notch and HH pathways induce CSCs phenotypes. Notch-Hedgehog positive tumor cells mediate immune evasion by enhancing the functions of regulatory T cells (79).

### HH Pathway and the Tumor Microenvironment in Lung Cancer

The tumor microenvironment, a local homeostatic environment composed of tumor cells, stromal cells (including fibroblasts, immune and inflammatory cells) as well as the extracellular matrix, provides the necessary materials for tumor initiation and progression (80). Forkhead box F1(FoxF1) is associated with poor prognostic outcomes in some lung cancer subtypes. FoxF1, a downstream effector of HH signaling, stimulates the secretions of HGF and FGF2 by lung cancer fibroblasts, promoting lung cancer cell growth and migration *in vivo* (81).

NSCLC cells activate HH signaling in lung fibroblasts in a paracrine manner to stimulate the production of pro-angiogenic and metastatic factors, which induce fibroblast proliferation, invasion and collagen deposition, leading to lung cancer progression (82). Similarly, tumor cell-derived HH ligands stimulate perivascular stromal cells to secrete VEGF-A, promoting tumor angiogenesis *in vitro* and *in vivo* (83). CAFs-derived SHH ligands activate HH signaling in NSCLC cells in a paracrine manner, upregulating the expressions of EMT-related genes such as α-SMA and FAP, and enhancing the migration abilities of lung cancer cells (84). CAFs remodel actin cytoskeleton-induced EMT process in an Smo-dependent manner, enhancing the insensitivity of lung cancer cells to EGFR-TKIs (85).

TAMs receiving HH ligands secreted by tumor cells activate KLF4 and STAT3, downstream transcription factors of HH signaling, which drive TAMs M2 polarization, reduce CD8+ T cells, and inhibit their functions, thereby promoting malignant progression in various tumors (86, 87). By co-culturing A549 cells with THP-1-derived macrophages, A549 cells stimulated THP-1-derived macrophages toward TAMs M2 polarization. In contrast, THP-1-derived macrophages upregulate the expressions of stemness-related genes, including Sox2 and NANOG through HH, STAT3 and Notch signaling, increasing the stemness characteristics of lung cancer CSCs (88).

In conclusion, CAFs and TAMs interact with tumor cells to induce a pro-tumorigenic microenvironment, and HH signaling in the tumor microenvironment regulates CAFs, TAMs, and tumor cells in an autocrine and paracrine manner to increase stemness characteristics and promote the EMT process. Targeting HH signaling is a promising strategy to inhibit the formation of the tumor microenvironment.

## HH Pathway and Lung Cancer Prognosis

It has not been conclusively determined whether HH pathway-related proteins are associated with lung cancer prognosis. We reviewed recent findings regarding the biological functions of the HH pathway in lung cancer, to establish the link between HH signaling proteins and prognostic outcomes of lung cancer patients (**Table 1**).

**Table 1:** The correlation between HH signaling proteins and the prognosis of lung cancer.

Sample	Pathology subtype	TNM	Method	Gene or Protein	Prognostic marker	Reference
167	LSCC, LAC	I-IV	IHC	SHH	SHH	(89)
403	LSCC, LAC	I-IV	IHC	Gli3FL, Gli3TR	Gli3TR	(90)
40	LSCC, LAC	I-II	IHC	SHH, Gli1, LYVE-1, VEGF-D	SHH, Gli1, LYVE-1	(91)
248	LSCC, LAC	I-II	IHC	SHH, Ptch1, Smo, Gli1, Gli2, ALDH1A1	NO	(92)
81	LSCC, LAC	I-IV	IHC	SHH, Ptch1, Smo, Gli1,	NO	(93)
101	LSCC	I-III	IHC	Gli1, LSD1, CD44, Sox9, Gli1, Sox2	Gli1	(94)
102	LAC	II-IV	qPCR	Gli1mRNA, Gli2mRNA, Gli3mRNA	Gli1mRNA	(95)
166	LAC	I-IV	IHC	SHH, Gli1, Gli2, Gli3, ABCG2	NO	(96)
12	SCLC	III-IV	qPCR	Gli1mRNA	Gli1mRNA	(97)
36	SCLC	III-IV	IHC	SHH, Gli1, Ptch1, Smo	SHH	(98)

Hwang et al. (91) performed immunohistochemistry (IHC) to assess the expressions of SHH, Gli1, LYVE-1, and VEGF-D in 40 cases of primary NSCLC tissues. HH signaling protein overexpression, especially SHH, represent an independent risk factor for NSCLC. Moreover, SHH protein overexpression is negatively correlated with tumor differentiation and predicts poor prognostic outcomes in LSCC patients (89).

Gli proteins are potential CSCs markers and independent prognostic factors in LSCC. Their overexpression is closely associated with various malignant behaviors of LSCC, including TNM staging, lymph node metastasis, and clinical staging (94). Ishikawa et al. (95) measured the expression levels of Gli1, Gli2, and Gli3 mRNA in surgical samples from stage II-IV LAC patients using q-PCR. Their findings implied that Gli1 mRNA can serve as potential independent biomarker for prognosis in advanced LAC patients. Moreover, Lim et al. (98) evaluated the expression levels of HH signaling-related proteins, including SHH, Ptch, Smo, and Gli1, in extensive-stage SCLC samples. Their data revealed that the other markers have nothing to do with patient prognosis, while SHH proteins are potential markers for PFS and OS. Additionally, it has also been reported that truncated Gli3 (Gli3TR) is essential for LAC initiation and acts as a potential prognostic risk factor (90).

Overexpression of Gli1 tends to imply progressive stages and is associated with unfavorable prognostic outcomes (89, 99). In contrast, Gli1 mRNA overexpression is associated with better survival outcomes for advanced SCLC patients (97). Savani et al. (93) assayed tissue microarray with 81 samples from 42 patients with various NSCLC histologies. Minimum overexpression of HH signaling-related proteins in NSCLC do not correlate with patient outcomes. Additionally, there were no significant correlations between HH signaling-realted proteins, ALDH1A1 and RFS, OS in early-stage NSCLC (92). Kim et al. (96) showed that SHH and Gli1 overexpression imply better OS and PFS in LAC, but they are not independent prognostic factors.

## Recent Advances in HH Inhibitors

The three currently marketed HH inhibitors, Vismodegib and Sonidegib, are used to treat locally metastatic and advanced BCC (100–104), while Glasdegib is used for the treatment of acute myeloid leukemia (AML) (105, 106). Unfortunately, HH inhibitor resistance, which is attributed to both Smo acquired resistance and activation of noncanonical HH signaling, can never be avoided (107, 108). Therefore, there is an urgent need to develop new HH inhibitors to overcome HH inhibitor resistance and prolong the survival outcomes for patients with advanced HH-dependent tumors. **Table 2** shows the HH inhibitors currently in clinical trials for lung cancer.

**Table 2:** HH inhibitors in clinical trial, mainly focusing on lung cancer therapy.

Drug	Target	Number	Cancer Type	Clinical Trial	NCT trial	Status
Sonidegib	Smo	–	BCC	–	–	FDA
Vismodegib	Smo	–	BCC	–	–	FDA
Glasdegib	Smo	–	AML	–	–	FDA
Vismodegib (GDC-0449)	Smo	168	SCLC	Phase 2	NCT00887159	Completed
	Smo	67	Solid cancers	Phase 1	NCT00968981	Completed
	Smo	68	Unspecified adult solid tumor	Phase 1	NCT00607724	Completed
	Smo	63	Malignant neoplasm	Phase 1 Phase 2	NCT01174264	Completed
	Smo	31	Cancer	Phase 1	NCT01546519	Completed
	Smo	55	Adult solid neoplasm	Phase 1	NCT00878163	Active, not recruiting
	Smo	6452	Advanced malignant solid neoplasm	Phase 2	NCT02465060	Recruiting
Sonidegib (LDE225)	Smo	19	Lung cancer	Phase1	NCT01579929	Completed
	Smo	114	Advanced solid tumor	Phase 1	NCT01769768	Completed
	Smo	10	Ptch1 or Smo activated solid and hematologic tumors	Phase 2	NCT02002689	Terminated
	Smo	45	Advanced solid tumors	Phase 1	NCT01208831	Completed
	Smo	103	Advanced solid tumor cancers	Phase 1	NCT00880308	Completed
	Smo	30	Solid tumor	Phase 1	NCT01954355	Completed
PF-04449913	Smo	23	Solid cancers	Phase 1	NCT01286467	Completed
IPI-926 (Saridegib)	Smo	94	Neoplasms	Phase 1	NCT00761696	Completed
Itraconazole	Smo	17	NSCLC	Early phase 1	NCT02357836	Completed
	Smo	60	Lung cancer	Phase 1	NCT03664115	Unknown
Taladegib (LY2940680)	Smo	26	SCLC	Phase 1 Phase 2	NCT01722292	Terminated
	Smo	19	Neoplasm metastasis	Phase 1	NCT01919398	Completed
LEQ506		57	Advanced solid tumors	Phase 1	NCT01106508	Completed
BMS-833923		5	SCLC	Phase 1	NCT00927875	Completed
Arsenic Trioxide (ATO)	Gli	9	NSCLC	Phase 1	NCT02066870	Unknown
	Gli	30	Lung cancer	Phase 2	NCT00075426	Completed
	Gli	20	Lung cancer	Phase 2	NCT01470248	Completed
Simvastatin	Oxysterol	62	SCLC	Phase 2	NCT00452634	Completed
	Oxysterol	70	SCLC	Phase 2	NCT04698941	Not yet recruiting
	Oxysterol	110	Lung cancer	Phase 2	NCT00452244	Completed
	Oxysterol	192	SCLC	Phase 2	NCT01441349	Recruiting
	Oxysterol	84	NSCLC	Phase 2	NCT01156545	Unknown

Itraconazole, a classic antifungal drug, has excellent pharmacological characteristics and safety. It acts on Smo proteins with a site of action distinct from that of Vismodegib, exhibiting potent anti-HH signaling effects (109–111). In a phase 2 trial involving metastatic nonsquamous NSCLC, itraconazole combined with pemetrexed significantly prolonged the overall survival outcomes for patients by up to 32 months, compared to pemetrexed alone (8 months) (112). In a randomized controlled study investigating the effects of itraconazole on clinical outcomes in patients with advanced NSCLC receiving platinum-based chemotherapy, it was confirmed that itraconazole significantly improved 1-year PFS and overall response rates (ORR), but not 1-year OS (113). The rationale for these two studies was based on anti-angiogenic effects of itraconazole. HH signaling is highly associated with tumor angiogenesis, however, a limited number of studies have determined whether itraconazole affects tumor angiogenesis through HH signaling. In advanced SCLC patients, the Smo inhibitor, Sonidegib, combined with standard chemotherapy, improved clinical outcomes were well tolerated. In one case of advanced SCLC with Sox2 amplification, progression free survival after combination therapy was as long as 27 months (114).

In conclusion, HH inhibitors alone or combined with other agents, including chemotherapeutic agents and small molecule targeted agents, have significant therapeutic implications in HH-dependent lung cancer patients. The current clinical trials have some limitations. First, there was no patient stratification before clinical trials; Second, activation mechanisms of canonical and non-canonical HH signaling in lung cancer are poorly understood; Third, there is a lack of large samples, multicenter data for further validation.

### HHat Inhibitors

Through autoproteolytic cleavage, full-length SHH proteins generate 25 kDa SHH-C and 19 kDa SHH-N. Then, SHH-N undergoes C-terminal cholesterylation and N-terminal palmitoylation to mature into the SHH-N ligand (22). HHat mediates N-terminal palmitoylation of SHH-N and is essential for SHH-N ligand maturation. HHat inhibitors inhibit SHH-mediated activation of canonical and noncanonical HH pathways by blocking SHH-N ligand palmitoylation. RU-SKI43 (IC_50_: 0.85 μM), a small molecule inhibitor, exhibited HHat inhibitory effects *in vitro and in vivo* (115). RU-SKI-201 (IC_50_: 0.73 μM), optimized based on RU-SKI43, overcame the off-target toxicity of RU-SKI 43 and specifically inhibited HH signaling in multiple tumor cell lines (116). Although both small molecule inhibitors exhibit potent anti-HH signaling activities, their oral availability and pharmacological toxicities should be evaluated at the animal level.

### SHH Inhibitors

The inhibition of HH proteins induces all Ptch-induced pro-apoptotic pathways (117). 5E1, a monoclonal antibody to SHH-N, has been widely used in biological experiments. Interestingly, most of the binding sites of 5E1 on SHH-N ligands overlap with HHIP (118). Similar to 5E1, Tolani et al. developed a new therapeutic antibody that targets the full-length SHH protein and SHH-N ligand to inhibit tumor growth (119). Owens et al. identified HL2-m5 (IC_50_: 230 nM), a macrocyclic peptide inhibitor with a high affinity for SHH ligands, which inhibited HH signaling *in vitro* without significant pharmacological toxicities (120). This compound should be further validated *via* animal experiments to assess its oral availability and safety.

### Smo Inhibitors

Smo inhibitor resistance is the leading cause of progression in most HH-dependent tumor patients and is mainly attributed to mutations in the drug binding pocket of the Smo protein (121). In a phase I trial, patients with locally metastatic and progressing BCC previously treated with, or not with HH inhibitors exhibited good clinical responses to LY2940680 (Taladegib) and were well tolerated (122). Cyclopamine and its derivatives from natural compounds reverse HH signaling activation by targeting Smo, thereby limiting HH-dependent tumor cell growth (123). As a semisynthetic cyclopamine analogue, IPI-926 (Saridegib) addresses the issues of low potency and poor aqueous solubility of cyclopamine. Significantly, it inhibited tumor progression in HH-dependent medulloblastoma (MB) mice models with no obvious toxicity (124). IPI-926 has certain inhibitory activities against Smo^D473H^ (Smo mutant), which is resistant to GDC-0449 (Vismodegib). When combined with gemcitabine, IPI-926 was shown to enhance anti-tumor activities of gemcitabine by increasing tumor vascular densities, which prolonged the survival outcomes of mice with pancreatic ductal adenocarcinoma (125). In a clinical phase I study involving basal cell carcinoma patients, despite differences in chemical structures between IPI-926 and Vismodegib, both exhibited similar sites of action on the Smo protein and shared same resistance mechanisms (126).

The FDA approved itraconazole for antifungal treatment, and its safety as well as side effects are well understood (111, 127). Compared to Vismodegib, Itraconazole inhibits Smo through different mechanisms. Itraconazole exerts significant anticancer activities *in vivo*, even targeting Vismodegib-resistant Smo^D477G^ (110). Similar to itraconazole, posaconazole exerts anti-tumor activities by targeting Smo. Interestingly, posaconazole without triazole retains its antagonistic effects on HH signaling and disrupts the inhibitory effect on Cyp3A4 (128). The des-triazole derivatives, based on the posaconazole scaffold, were effective at inhibiting HH signaling and were well tolerated (128).

The presence of an oxysterol binding site in each of the seven transmembrane domains and extracellular cysteine-rich domain (CRD) of the Smo protein is necessary for HH signaling activation (129, 130). Cholesterol molecules can be transferred from the cytoplasm to oxysterol sites on the seven transmembrane regions or extracellular CRD *via* hydrophobic channels on Smo proteins, mediating Smo activation (129, 130). Therefore, the oxysterol binding site on the Smo protein is a potential HH signaling target. There is a need to determine the mechanisms through which the Smo protein integrates the information conveyed by the two oxysterol binding sites to determine the activities of HH signaling *in vivo*. As a Bcl-2 inhibitor, ABT-199 is approved by the FDA for treatment of chronic lymphocytic leukemia (ALL). ABT-199 (IC_50_: 215.9 nM) targets the extracellular CRD of Smo while acting as a potential competitive inhibitor of oxysterol, which inactivates HH signaling (131). In addition, ABT-199 was shown to target the drug binding pocket mutant and/or constitutively activated Smo proteins with excellent pharmacological properties and safety, therefore, its effects in patients with HH inhibitor resistance should be evaluated (131).

Multi-targeted HH inhibitors act on multiple components of HH signaling and/or inhibit multiple noncanonical HH pathways, exhibiting potent inhibitory anti-HH signaling effects. Lospinoso Severini et al. identified compound 22 (IC_50_: 7.1 μM), which contains an isoflavone scaffold, to be a multi-target HH inhibitor that inhibits MB cell growth by targeting Smo and Gli proteins (132). As a semisynthetic oxysterol analogue, Oxy210 (IC_50_: 1.83 μM) is a dual inhibitor of TGFβ and HH signaling, with excellent pharmacological properties, but should be further evaluated at the animal level (133). Li et al. and Zhu et al. determined that HH-13, HH-20 (IC_50_: <0.2 μM), and L-4 (IC_50_: 2.33 nM) act as potential Smo inhibitors by targeting Smo^D473H^, respectively (134–136). Moreover, L-4 showed high efficiency, good tolerability, and high oral bioavailability in ICR mice (134).

In conclusion, reliable HH inhibitors from FDA-approved drugs, such as itraconazole and ABT-199 have been screened and their toxic effects as well as side effects evaluated. The Oxysterol site of the Smo protein is essential for activation of HH signaling, and the development of Smo inhibitors, based on the Oxysterol site, can overcome Smo acquired resistance, with great potential for clinical applications.

### Gli Inhibitors

Gli inhibitors target Smo downstream genes to overcome Smo acquired resistance and inhibit Gli-dependent noncanonical pathways, thereby achieving better clinical efficacies and improving patient clinical outcomes. GANT58 and GANT61 (IC_50_: 5 μM) are widely used in biological experiments by targeting the Gli1 transcription factor (137). Notably, GANT61 is more active against HH signaling than GANT58, which is mainly attributed to its ability to interfere with the binding of Gli1 to DNA (137). The Gli-activated but Smo-independent pathway is activated in some types of LSCC, including PI3K/AKT and RAS-MEK signaling. Therefore, GANT61 is more potent than Vismodegib in Gli-dependent LSCC patients (138).

Arsenic trioxide (ATO) (IC_50_: 2.7 μM), approved by the FDA for acute promyelocytic leukemia (APL), targeted the Gli1 transcription factor and exhibited significant cytotoxicity in mice models of MB and Ewing sarcoma, supporting further pharmacological evaluation in Gli1-dependent malignancies (139). ATO kills SCLC CSCs by downregulating CSCs-related genes, such as Sox2 and c-Myc at least in part by targeting HH signaling (140). The lack of efficacy of ATO in relapsed SCLC patients and SCLC patient-derived xenografts (PDX) mice in phase II clinical trials may be attributed to low proportions of SCLC CSCs and intermittent dosing regimens (141). The therapeutic benefits of ATO in combination with chemotherapeutic agents and daily continuous dosing regimens in SCLC patients should be investigated (141). Due to differences in study methods and different cell lines, it should be determined if the primary target of ATO is Gli1 or Gli2 (142). Pyrvinium (IC_50_: 10nM) is an FDA-approved casein kinase-1a (CK1a) agonist with high efficacies. Moreover, CK1a can also act as a negative regulator of HH signaling (143). Mechanistically, CK1a downregulates HH target genes by modulating their stability *via* phosphorylation of Gli transcriptional factors (143).

Studies have found that VDR signaling negatively regulates Gli1 expression through crosstalks with HH signaling (144–148). Vitamin D3-based derivative compounds 16, 21, and 22 modulated Gli1 proteins *in vitro* by targeting VDR signaling and HH signaling (149). However, the mechanisms through which VDR regulates HH signaling have not been elucidated, which requires further investigations.

Bromodomain (BRD), a class of conserved protein domains that specifically recognize acetylated lysine in histones, promote the enrichment of chromatin remodeling factors and transcription factors to specific gene transcription sites by binding acetylated lysine, altering the activities of RNA polymerase II, and regulating gene expressions (150, 151). Liu et al. developed non-selective BET inhibitors, compounds 25 and 35 (IC_50_: < 1nM) by further optimization of the BRD4 inhibitor, ABBV-075, which inhibited Gli protein expressions and overcame resistant Smo mutations in MB mice without apparent toxicity (152). Compared to compound 35, compound 25 exhibits stronger efficacies and better safety, however, it should be optimized to improve its pharmacokinetic parameters (152).

In patients with recurrent metastatic BCC, Itraconazole in combination with ATO (ATO-ITRA) reduced Gli1 mRNA levels by 75% from baseline levels (153). Three of the five patients treated with an ATO-ITRA regimen were stable for three months, but did not achieve tumor shrinkage, which was attributed to sequential dosing as well as lower doses. Higher doses and/or daily continuous dosing regimens should be used to evaluate the clinical efficacy of the ATO-ITRA regimen (153).

**Figure 5:**
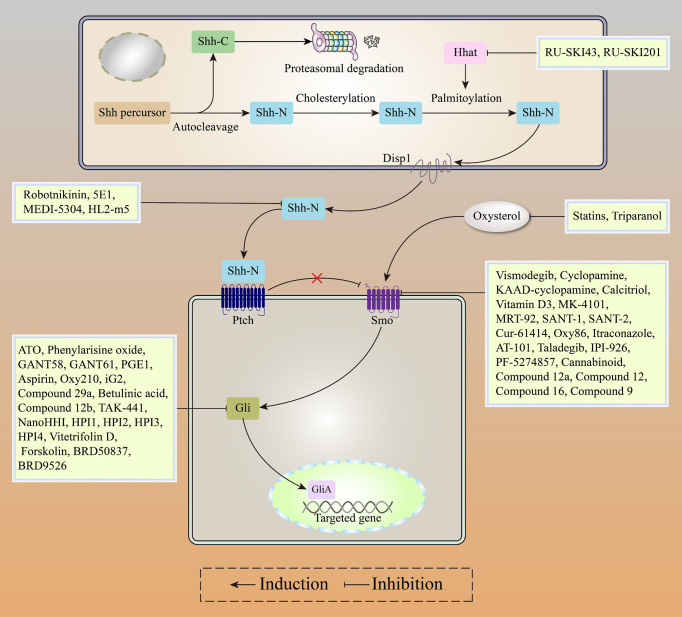
Recent advances in HH inhibitors, including HHat inhibitors, SHH inhibitors, Smo inhibitors, and Gli inhibitors.

In conclusion, Gli inhibitors overcome Smo acquired resistance and inhibit Gli activatation *via* the Smo-independent noncanonical pathway, with robust anti-tumor activities in Gli-dependent tumors. Gli inhibitors (including ATO and GANT61) combined with Smo inhibitors (including Itraconazole and Taladegib) or chemotherapeutic agents represent a promising strategy for patients with HH-dependent tumors (**Figure 5**).

## The HH Pathway and Natural Products for Lung Cancer Therapy

**Figure 6:**
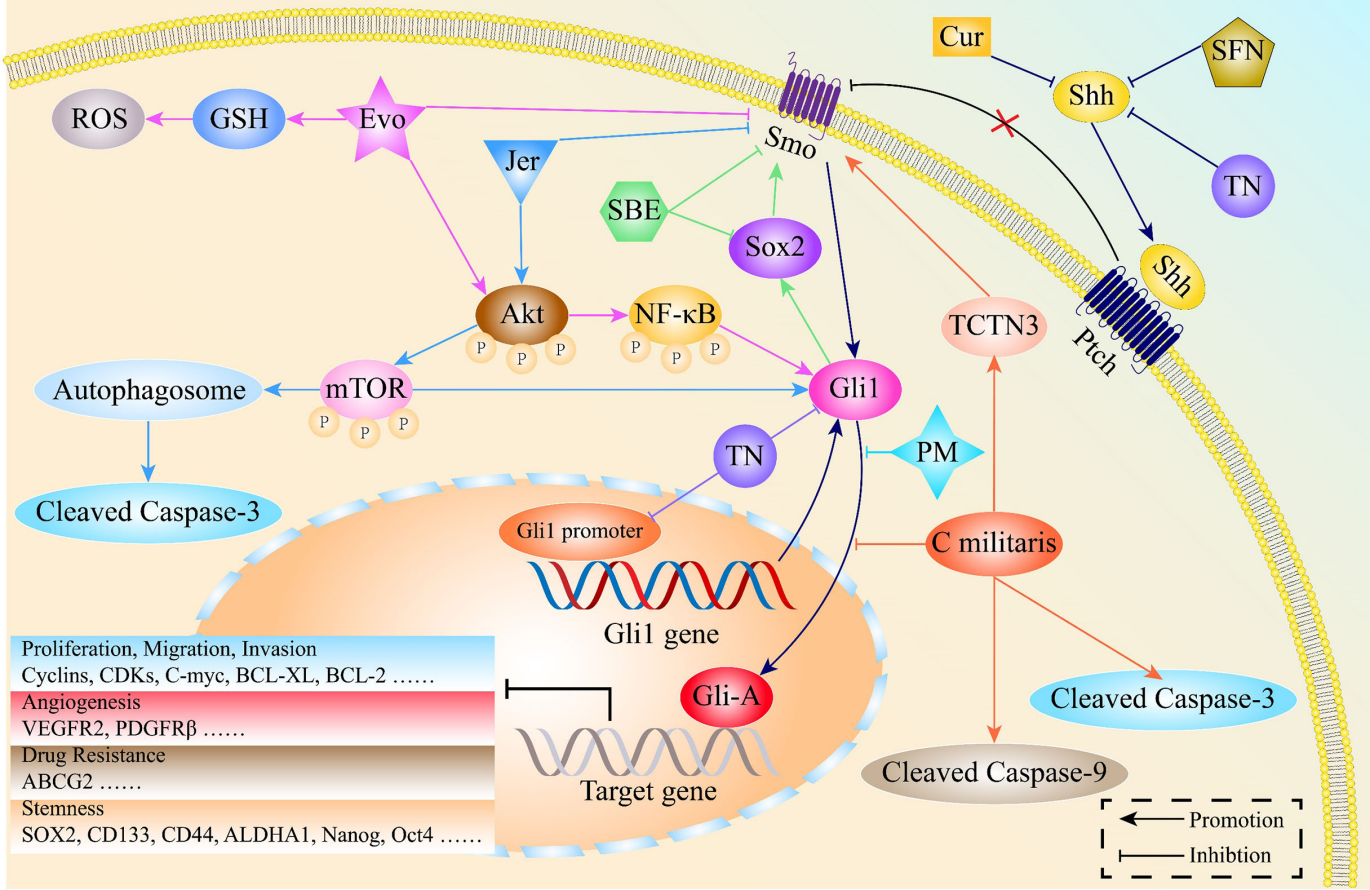
The mechanisms through which natural products exert potent anti-cancer activities by regulating the HH pathway in lung cancer. Jervine (Jer), Evodiamine (Evo), Scutellariabarbata D. Don extraction (SBE), Sulforaphane (SFN), Triptonide (TN), Curcumin (Cur), Pristimerin (PM), Cordyceps militaris Exerts (C militaris), Tectonic protein 3 (TCTN3).

Apart from being easily accessible, natural products have potent anti-inflammatory, anti-bacterial as well as anti-tumor activities (154, 155). Natural products inhibit cell proliferation, invasion, EMT processes as well as stemness features by targeting HH signaling in various malignancies, including lung cancer [**Figure 6** (156, 157)].

### Natural Products Induce Cell Apoptosis in Lung Cancer by Inhibiting the HH Pathway

Autophagy, a process of cellular self-degradation, removes damaged or redundant proteins as well as organelles. It plays an important role in maintaining intracellular homeostasis as well as tumorigenesis (158–161). Autophagy is a double-edged sword that promotes and inhibits tumor development (162–164). Lei et al. reported that Jervine triggers autophagy and increases the expressions of cleaved caspase 3, leading to NSCLC cell apoptosis by acting on AKT/mTOR and HH pathways (165).

The additional natural products that block cell proliferation include Evodiamine, Sulforaphane, and Cordyceps militaris. Evodiamine inhibits cell proliferation by modulation of the AKT/NF-κB and HH pathways in NSCLC (166). Cordyceps militaris induces NSCLC cell apoptosis by blocking Gli1 nuclear translocation *via* inhibiting the expressions of tectonic protein 3 (TCTN3) (167). In the PC9 cell line, Sulforaphane reversed lung cancer cell resistance to gefitinib by regulating HH signaling (168).

### Natural Products Suppress Lung Cancer Cell Invasion and Metastasis by Inhibiting the HH Pathway

As a traditional Chinese medicine, Scutellariabarbata D. Don extraction (SBE)has been used in clinical management for many years because of its reliable and effective anti-cancer activities (169, 170). SBE downregulates the downstream components of HH signaling, leading to tumor cell cycle arrest and increasing lung cancer cell sensitivity to cisplatin (171).

Tumor angiogenesis is closely associated with multiple malignant behaviors of lung cancer, such as proliferation, invasion, and metastasis. Inhibition of tumor angiogenesis is of great clinical significance. However, angiogenesis inhibitors that target VEGF/VEGFR2 did not significantly improve the five-year survival rate for cancer patients (172, 173). Therefore, there is a need to develop novel angiogenesis inhibitors that lack toxic effects. Pristimerin, a compound isolated from Celastrus aculeatus Merr, exhibits multiple pharmacological activities, including anti-inflammatory, anti-microbial, anti-tumor, and anti-peroxidative effects (174, 175). In NSCLC, Pristimerin affects the maturation of tumor angiogenesis by regulating the translocation of its downstream gene (Gli1) to the nucleus through HH signaling (176).

### Natural Products Eliminate Lung Cancer CSCs by Inhibiting the HH Pathway

Triptonide, a natural compound separated from Tripterygium wilfordii Hook, exhibits potent anti-inflammatory (177) and anti-tumor efficacies (178, 179). It has been shown to inhibit the proliferation, invasion, as well as migration of lung cancer cells and eliminates stem-like signatures by reducing the expression levels of Gli1 at both transcriptional and translational levels *via* inhibiting Gli1 promoter activities (180).

Curcumin is a potent compound isolated from the rootstalk of Curcuma longa (181, 182). It was shown to effectively eliminate various cancer stem cells and reduce their CSCs-like characteristics (183–185). Curcumin has powerful inhibitory effects on lung cancer CSCs *in vitro*. Mechanistically, Curcumin downregulates the expression levels of CSCs markers, including CD133, CD44, ALDHA1, Nanog and Oct4, by inhibiting the HH and Wnt/β-catenin pathways (186). In addition, SBE suppresses stemness-like phenotypes in NSCLC by targeting the Sox2/Smo/Gli1 positive feedback loop (187).

## Conclusions and Future Directions

The HH pathway plays a pivotal role in lung cancer oncogenesis and development. The HH pathway is involved in drug resistance and CSCs. In resistant-EGFR-TKIs lung cancer cells, aberrant activations of the HH pathway are frequently observed. Therefore, the combination of HH inhibitors and EGFR-TKIs provide a novel treatment strategy for advanced lung cancer patients.

The noncanonical HH pathway, especially the Gli-activated but Smo-independent signaling, promotes the malignant progression of lung cancer, including cell proliferation, invasion, migration, metastasis, EMT and stemness features. Thus, it is necessary to block the noncanonical HH signaling in patients with HH-dependent tumors.

Additionally, we summarized recent findings regarding the molecular mechanisms of the HH pathway in promoting lung cancer progression and discussed the correlation between HH pathway-related proteins and prognostic outcomes for lung cancer patients. HH pathway-related proteins have been shown to predict prognostic outcomes in advanced NSCLC patients, and overexpressions of SHH and Gli1 are strongly associated with poor prognostic outcomes. Therefore, studies should focus on prognostic values of HH pathway-related proteins in patients with late-stage lung cancer.

Studies are aimed at developing novel Smo and Gli inhibitors. However, although Vismodegib and Sonidegib have shown good efficacies in clinical treatment, they remain at risk of drug resistance, which is mainly attributed to mutations in the drug binding pocket of the Smo protein. Itraconazole exerts potent anti-tumor activities in Vismodegib-resistant tumors because it has a different active site on Smo than Vismodegib. Furthermore, the safety, pharmacokinetics and toxicity of itraconazole as a traditional antifungal drug are well understood, resulting in significant cost saving in drug development. The clinical efficacy and toxicity of Itraconazole in treatment of lung cancer has been assessed. A Phase II study conducted by Rudin et al. investigated the implications of itraconazole in combination with pemetrexed in lung cancer therapy. The results showed that itraconazole significantly prolonged the overall survival outcomes of patients by up to 32 months, compared to pemetrexed alone (8 months), with mild adverse effects. Notably, the preclinical trial was designed based on the principle that itraconazole inhibits tumor angiogenesis. However, it was not determined whether itraconazole affects malignant progression by inhibiting lung cancer angiogenesis through the HH pathway. By targeting the downstream transcription factors of HH signaling, Gli inhibitors overcome Smo mutations and block Gli-activated but Smo-independent noncanonical signaling, with promising clinical applications in HH-dependent tumors. ATO, an FDA-approved drug for APL treatment, exerts potent antitumor activities by targeting Gli1/2 proteins and has excellent pharmacological properties. Itraconazole, in combination with ATO, significantly downregulated Gli1 mRNA expression levels and prolonged PFS in BCC patients (3 months). However, due to sequential dosing and lower doses, the ATO-ITRA regimen did not downsize the tumor. Studies on ATO-ITRA may allow us to test higher doses or continuous dosing regimens to assess clinical efficacies in HH-dependent tumor patients.

In addition, some natural compounds have been used in oncology treatment for many years, and are characterized by wide sources, low-toxicity and broad-spectrum anti-tumor properties. A phase II trial of whether curcumin in combination with Lovaza (made with fish oils) reduces the sizes of lung nodules will be conducted soon. Interestingly, despite the general disadvantages of poor oral bioavailability and low potency of some natural compounds such as curcumin, researchers have developed a number of nano-delivery systems for curcumin, which have greatly improved its aqueous solubility and anti-tumor potency. In addition, various natural compounds can act on multiple targets and are involved in extensive oncogenic signaling networks, which should be investigated further. In future, more natural extracts will be developed, but further optimization and modification for these compounds to enhance their anti-tumor activities, oral bioavailability and reduce drug toxicity will be a great importance.

## Author Contributions

Z-GS and NZ designed the work. CM wrote the manuscript. KH and IU prepared the figures and tables. Q-KZ drafted and revised the manuscript. All authors read and approved the final manuscript.

## Funding

This work was supported by the Shandong Provincial Natural Science Foundation (grant no. ZR2020MH204), the 19th batch of science and technology innovation development plan of Jinan in 2020 (Clinical medicine science and technology innovation plan, grant no.202019032), and the second group of science and technology projects of Jinan Health Committee (grant no. 2020-3-15).

## Conflict of Interest

The authors declare that the research was conducted in the absence of any commercial or financial relationships that could be construed as a potential conflict of interest.

## Publisher’s Note

All claims expressed in this article are solely those of the authors and do not necessarily represent those of their affiliated organizations, or those of the publisher, the editors and the reviewers. Any product that may be evaluated in this article, or claim that may be made by its manufacturer, is not guaranteed or endorsed by the publisher.
